# Factors Associated with Feeding Problems in Young Children with Gastrointestinal Diseases

**DOI:** 10.3390/healthcare9060741

**Published:** 2021-06-17

**Authors:** Katerina Sdravou, Elpida Emmanouilidou-Fotoulaki, Athanasia Printza, Elias Andreoulakis, Stavroula Beropouli, Giorgos Makris, Maria Fotoulaki

**Affiliations:** 1 4th Department of Pediatrics, School of Medicine, Aristotle University of Thessaloniki, General “Papageorgiou” Hospital, 56403 Thessaloniki, Greece; elpisemfot@hotmail.com (E.E.-F.); mfotoul@otenet.gr (M.F.); 2 1st Otolaryngology Department, School of Medicine, University Hospital AHEPA, Aristotle University of Thessaloniki, 54636 Thessaloniki, Greece; aprintza@auth.gr; 3 Hellenic Centre for Mental Health and Research, Department of Thessaloniki, Adult Psychiatric Unit, 36 Kaftatzoglou Str, 55337 Thessaloniki, Greece; eliasandreoulakis@gmail.com; 4 Department of Pediatrics, General Hospital of Kozani (Mamatseio), 1 K. Mamatsiou, 50100 Kozani, Greece; sberopouli@gmail.com; 5 Department of Speech and Language Therapy, School of Health Sciences, University of Peloponnese, 2400 Kalamata, Greece; glog_makris@yahoo.gr

**Keywords:** feeding problems, children, gastrointestinal diseases, mealtime environment, parental feeding practices, risk factors

## Abstract

Feeding problems are associated with the consumption of a limited amount or restricted variety of foods and often occur in children with gastrointestinal diseases. The majority of studies to date do not use valid and reliable measurements to detect feeding problems. The aim of this cross-sectional study was to assess behavioral and skill-based feeding problems in young children with gastrointestinal diseases by using a well-established parent-reported feeding measure and identify demographic, anthropometric, and environmental factors associated with maladaptive feeding behaviors in this pediatric population. Parents completed the Greek version of the Behavioral Pediatrics Feeding Assessment Scale (BPFAS) and self-reported questionnaires assessing mealtime environment and parental feeding practices. It was found that 18.6% of the sample had abnormal Total Frequency Score (TFS) (frequency of problematic feeding behaviors) and 39.5% had abnormal Total Problem Score (TPS) (number of behaviors perceived as problematic by parents). Younger children, with lower body mass index, lower birth weight, and only children were more likely to have feeding problems. The study showed that parent-reported feeding problems are increased in young children with gastrointestinal diseases and are associated with specific aspects of mealtime environment and parental feeding practices.

## 1. Introduction

Feeding problems in young children are common, affecting almost one third of typically developing children and up to 80% of children with developmental disabilities [1,2]. Numerous studies have shown that children with gastrointestinal diseases, especially children with gastroesophageal reflux and eosinophilic esophagitis, face higher risk of developing feeding problems [3]. Feeding problems may have several organic, developmental, psychological, and sociological implications. Even when growth and nourishment remain unaffected, the life quality of both family and child can be seriously degraded [4]. The spectrum of feeding problems varies from selectivity, poor appetite, feeding skills deficits to complete food refusal, and often involves a combination of interrelated problems [5]. Feeding problems in most cases arise because of a complex interplay of organic and environmental influences on a child’s feeding behavior [6,7,8]. To date, there is strong evidence to suggest that environmental factors, such as unfavorable mealtime environment and negative parental feeding practices, are associated with the development or maintenance of feeding problems in early childhood [9,10,11,12,13,14]. These factors are highly significant for children with gastrointestinal diseases, who not only face the risk of developing feeding problems, but they might also be asked to strictly follow a challenging treatment comprised of limitations in diet or special diet resulting in greatly influencing child and parent feeding behavior [15,16]. Recent evidence supports that mealtime environment in children with gastrointestinal diseases significantly deviates from that of healthy children [17]. Parents use a significantly more often inappropriate feeding practices in these populations [18]. Yet, no data exist on the association between environmental factors and feeding problems in children with gastrointestinal diseases.

The complexity of feeding issues has led to an inconsistency both to the definition of feeding problems and the evaluation methods utilized in previous studies [5,19]. Therefore, it is challenging for feeding problems to be identified, prevented, and treated efficiently. One common definition of feeding problems is the inability or the refusal to eat certain foods or adequate amount of food. Only recently, a consensus definition has been proposed, according to which “Pediatric Feeding Disorders are defined as impaired oral intake that is not age-appropriate, and is associated with medical, nutritional, feeding skill, and/or psychosocial dysfunction” [19]. The majority of the existing studies used sets of questions instead of reliable and valid questionnaires to assess feeding problems in young children or focused only on specific behaviors such as selectivity. Yet, a very limited number of studies have used trustworthy measures of assessment and description of feeding problems in children with gastrointestinal diseases. Thus far, the Behavioral Pediatrics Feeding Assessment Scale (BPFAS) is regarded to be the most reliable and valid parent-administered feeding questionnaire [20,21]. This questionnaire covers a wide span of feeding difficulties namely food refusal, food selectivity, and oral motor/swallowing problems. Wu et al. used the BPFAS to assess feeding problems in 92 children with eosinophilic gastrointestinal disorders [22]. The authors found that those children had greatly higher levels of feeding problems in comparison with the control group. Mehta et al. investigated feeding problems, growth and nutrition in 91 children aged 1–7 years with gastroesophageal reflux disease (GERD) and eosinophilic esophagitis using the BPFAS [23]. The results showed that children with GERD and eosinophilic esophagitis had higher levels of problematic feeding behaviors than healthy children and almost one third of the clinical groups presented abnormal scores. Despite the fact that feeding problems are commonly encountered in children with gastrointestinal food allergies [24], the existent literature does not offer enough evidence to establish a firm connection between feeding problems and the specific gastrointestinal conditions [3]. Previous research was mainly relying on retrospective chart reviews of children with gastrointestinal food allergies [25,26,27,28].

In an effort to address the aforementioned limitations in the current literature, the aims of this study were the following: (i) to assess feeding problems among young children with gastrointestinal diseases in a sample with high representation of gastrointestinal food allergies using the BPFAS and (ii) to examine whether feeding problems are associated with mealtime environment, parental feeding practices, demographic and anthropometric characteristics in children with gastrointestinal diseases.

## 2. Materials and Methods

The Bioethics and Ethics Committee of the medical school of the Aristotle University of Thessaloniki gave approval for this cross-sectional study. All parents who took part in the study gave written informed consent.

### 2.1. Participants

The participants were the parents of 141 children (every child represented by one parent) with gastrointestinal diseases. Inclusion criteria comprised children aged 2 to 7 years old, with native Greek parents and diagnosed with a gastrointestinal disease by a pediatric gastroenterologist. The most commonly diagnosed diseases during the recruitment period for this age group were food allergies or intolerances (mainly cow’s milk protein allergy) with digestive manifestations such as enteropathy, colitis, and GERD. Children with developmental neurological disorders or chronic diseases possibly affecting their feeding behavior or their swallowing motor pattern (for example prematurity) were excluded from the study. The participants were patients of a pediatric gastroenterology outpatient clinic. Parents were explicitly informed that the utilization of the data for research was not interrelated with access to the service. Parents had the flexibility to complete the questionnaires at home or not complete them at all. The recruitment procedure took place from November 2016 until June 2018.

### 2.2. Measures

#### 2.2.1. Demographic and Anthropometric Data

The demographic characteristics of both parents and children such as gender, age, educational level, employment status, siblings, birth order as well as anthropometric measures such as birth weight, current height, and current weight were recorded. The conversion of height and weight scores to age and gender specific BMI z-scores was conducted with the World Health organization’s Anthro [29] and Anthroplus [30] software.

#### 2.2.2. The Child’s Medical History

The medical history of the children was recorded through 13 questions most of which were of closed type, some were multiple choice, and two were open-ended. Four questions concerned the perinatal history (birth weight and type of delivery), two concerned the existence of feeding problems, and three the diseases possibly affecting feeding. One question was about having speech or attention deficit disorder and three about difficulties in gaining weight in the first year of life, following the first year and during the last term.

#### 2.2.3. Behavioral Pediatrics Feeding Assessment Scale (BPFAS)

Among the various psychometric tools used to detect feeding problems BPFAS is the most reliable and valid one [20,22,31,32,33,34,35,36]. It has been tested in different populations and age groups. It consists of two sections and 35 questions. The 25 questions of the first section assess the feeding behavior of the child and the 10 of the second section assess the parents’ feelings towards the child’s feeding patterns as well as the parental feeding practices. In each of the 35 questions parents must answer on the frequency a named behavior appears (on a 5-point scale from 1—never to 5—always) and whether this behavior is a problem for them (yes–no). This creates two separate scores, the total frequency score TFS (maximum score 175) and the total problem score TPS (maximum score 35), respectively. In case of getting a score higher than 84 for the TFS and higher than 9 for the TPS there is a risk of facing feeding problems. The present study used the Greek version of the BPFAS [37].

#### 2.2.4. Set of Questions Developed to Assess Parental Feeding Practices

The parental feeding practices were investigated via 23 Likert-type questions. Using a five-point scale from 1 (never) to 5 (always) parents rated the frequency they adopted a certain strategy when their child refused to eat or ate inadequately. Minimal intervention was marked with “Always” in the first two questions (“I accept that he/she may not be hungry, and I take the food away” and “I urge the child to eat with prompts”) while in the rest of the questions maximum intervention was marked with “Always”. Two speech and language therapists, a pediatric gastroenterologist and an otolaryngologist formed the team of feeding and swallowing specialists who determined the parental feeding practices assessed in the present study. More information about the selection of the included has already been described [38].

#### 2.2.5. Set of Questions Developed to Assess Mealtime Environment

A comprehensive set of 24 Likert-type questions was used for the investigation of specific aspects of mealtime environment such as family mealtime routine (3 items), mealtime structure (10 items), child’s intake control (3 items), parental intake control (4 items), and the parent–child communication about the child’s feelings of hunger or satiety (4 items). A 5-point Likert-type scale ranging from 1 (never) to 5 (always or almost always) was used to measure each item. The same team of specialists on feeding and swallowing problems defined the features of mealtime environment to be evaluated. More information on the construction of the relevant questionnaire has already been published [17].

### 2.3. Statistical Analysis

Descriptive statistics were calculated as appropriate. For continuous variables (i.e., child age, zBMI, birthweight, TFS, and TPS) means and standard deviations as well as medians and 1st (Q1) and 3rd (Q3) quartiles represented the summary statistics, whereas for categorical variables (other demographics, the answers to BPFAS items and the category where each participant would fall based on the TFS and/or TPS cut-off) the summary statistics included absolute frequencies and/or percentages. Normal distribution of raw TFS and TPS was assessed by means of a Kolmogorov–Smirnoff test took. Associations between the participants’ demographics and/or anthropometrics, on one hand, and the likelihood of a feeding problem (the latter indicated by abnormal TFS and/or TPS), on the other, were investigated by means of chi-square tests or Mann–Whitney’s U tests accordingly (i.e., for categorical and continuous variables, respectively). Finally, linear associations between raw TFS and TPS and the aforementioned continuous demographic and anthropometric variables of the study were investigated by calculating the Spearman’s Rho correlation coefficient. The statistical significance level (alpha) was set at 0.05. SPSS Statistics v20 statistical software (IBM, 142, Armonk, NY, USA) was used for the analysis.

## 3. Results

One hundred and forty-one parents of children with gastrointestinal diseases participated in the study. The diagnoses of the children are presented in Table 1, whereas descriptive statistics concerning the variables of the study are given in Table 2. The mean (average) TFS was 69.98 ± 17.89 and the average TPS was 8.93 ± 6.75. Taking into account the established cut-offs for TFS and TPS, 18.6% (according to TFS) to 39.5% (according to TPS) of the children exhibited problematic feeding behaviors that could be highly indicative of a feeding disorder.

According to the parental reports, the consumption rates of specific foods were the following: 77.2% consumed “very often” or “always” starches, 73.5% meat or fish, 59.3% milk, 56% fruits, and 56.5% vegetables. Certain unfavorable feeding behaviors on behalf of the child that were demonstrated with a relatively high frequency, i.e., “very often” or “always”, were reported as follows: food neophobia (unwilling to taste new food): 38.6%; decreased appetite: 24.3%; prolonged mealtimes (meal lasting more than 20 min): 38%; negotiation over eating (what to eat): 26.4%. Concerning the parents themselves, 55.8% of them reported that they were sure that their child ate enough, 61.5% stated they felt confident they could handle their child’s feeding behavior during mealtime and 19.4%, mentioned that they experienced feelings of anxiety or frustration during feeding their child. Some of the most common practices reported (i.e., “very often” to “always”) were preparing a different kind of food (22.2%) and coaxing (25.8%). The frequencies of the answers to the questions of the BPFAS scale are presented in detail in Table 3.

Associations between pathological BPFAS scores (TFS and TPS above the cut-offs) and demographic, anthropometric, and environmental variables were investigated. The results are shown in Table 4. In order to preserve readability only the conclusion concerning the association test are given, i.e., positive, negative, or none.

When TFS and TPS were treated as continuous variables (i.e., irrespective of the established cut-offs), the following were found: TFS was negatively associated with child age (rho = −0.280, *p* = 0.001), zBMI (rho = −0.218, *p* = 0.017), and birth weight (rho = −0.211, *p* = 0.016). TPS was negatively associated with the age of the child (rho = −0.284, *p* = 0.002). Regarding the mealtime environment, the strongest negative associations found with TFS or TPS were as follows: “My child eats autonomously (no adult help needed)” (rho = −0.533, *p* < 0.001 for TFS), (rho = −0.466, *p* < 0.001 for TPS); “My child eats the same food with the rest of the family” (rho = −0.452, *p* < 0.001 for TFS), (rho = −0.382, *p* < 0.001 for TPS); and “My child stays seated until the meal is over” (rho = −0.438, *p* < 0.001 for TFS) (rho = −0.344, *p* < 0.001 for TPS). Examining the correlation coefficients between TFS and TPS on one hand and the parental feeding practices on the other, the strongest positive associations found were as follows: “I urge the child to eat with prompts” (rho = 0.552, *p* < 0.001 for TFS), (rho = 0.492, *p* < 0.001 for TPS); “I customize the environment so that the child can eat” (rho = 0.550, *p* < 0.001 for TFS), (rho = 0.464, *p* < 0.001 for TPS); and “I ask my family or other people to encourage the child to eat” (rho = 0.516, *p* < 0.001 for TFS), (rho = 0.446, *p* < 0.001 for TPS).

## 4. Discussion

Feeding difficulties in childhood concern parents, and children with gastrointestinal diseases are at increased risk of developing feeding problems [3,16,23]. Although a more robust association between feeding problems and specific gastrointestinal conditions, such as gastroesophageal reflux disease (GERD) and eosinophilic esophagitis, has been established, there is still limited evidence to support this association with gastrointestinal food allergies. This study is the first to use a well-established parent-reported feeding measure in a sample with high representation of gastrointestinal food allergies and is also the first to explore the relationship between feeding problems and several aspects of mealtime environment and parental feeding practices in this pediatric population. Children with feeding problems in this sample were found to exhibit greater problematic feeding behaviors than those reported for healthy young children [39] including food neophobia (38.6%), decreased appetite (24.3%), prolonged mealtimes (38%), and negotiation over eating (26.4%). Despite the fact that these behaviors are often observed in children of this age, their frequency was found to be higher than those in typically developing children of the same age [39]. More specifically, the mean BPFAS scores of the current sample are significantly higher than those of a normative Greek sample [39]. When the established cut-offs were used, it was found that 18.6% and 39.5% of the sample had abnormal TFS and TPS, respectively. Both estimated prevalence rates (via TFS and TPS) in the present study are significantly higher than those reported for a sample of healthy, typically developing children, suggesting the need for high clinical suspicion in order to provide timely assessment and management decisions.

When compared to other gastrointestinal samples, both mean TFS (frequency of problematic feeding behaviors) and the percentage of children with abnormal TFS of the present study were found lower than that reported for children with eosinophilic esophagitis [22,23] and GERD [23]. This lower incidence of feeding problems in our study may be possibly attributed to the high representation of food-induced gastrointestinal allergy in our sample as eosinophilic digestive disorders and GERD are the main gastrointestinal causes for the manifestation of feeding problems [3]. Mean TPS (number of behaviors perceived as problematic by parents) of the present study and the percentage of abnormal TPS are nonetheless consistent with previous studies on children with eosinophilic esophagitis [22,23] and GERD [23]. The deviation between abnormal TFS and TPS percentages has been previously observed in a healthy pediatric sample [39] and is probably indicative of different sensitivity of the two scores depending on the “severity” of feeding problem. Abnormal TFS for instance may be indicative of more severe feeding difficulties whereas abnormal TPS may be more sensitive in detecting milder feeding problems. From a different perspective, the observed differences of the two scores may reflect the distance between objective description of feeding behaviors and its subjective interpretation by the parents as TFS concerns the record of a child’s behavior and TPS illustrates the parent’s evaluative judgment over this behavior (whether it is considered a problem for them or not).

The most common behaviors that concerned parents were vegetable consumption (35.1%) and food neophobia (42.3%). Considering that a correspondent increased frequency was found in healthy samples [39], these behaviors do not concern only children with gastrointestinal diseases. On the contrary, reduced appetite, prolonged meal duration, and oral-motor difficulties were more prevalent in the sample of this study compared to a healthy sample [39]. It is noteworthy that although children with primary oral-motor difficulties such as children with medical history of neurological disorders [40] and prematurity were excluded from the study, oral-motor difficulties were still prevalent in the children of our sample. More specifically, children quite often exhibited difficulty in chewing, ate only ground, strained or soft food, and had preference for liquids. This finding is consistent with the literature as numerous studies have demonstrated increased prevalence of oral motor difficulties in children with gastrointestinal diseases [41,42]. Τhis study aimed to record feeding problems in children whose feeding skills would normally had been acquired. By the age of two, children are expected to primarily feed themselves, to be able to chew a wide range of textures and handle feeding equipment. Prolonged mealtime was commonly reported by parents (38% reported meals over 20 min frequently or always) and has been described as a suggestive symptom of a feeding difficulty [43,44] possibly serving as a primary marker [45]. Moreover, record of meal length has been proposed as a mean to help pediatricians identify problematic feeding behaviors [46]. Prolonged mealtime has also been documented in children with gastrointestinal diseases [23] suggesting that specific problematic feeding behaviors may be related to the organic cause of the feeding difficulty. Furthermore, the high prevalence of limited appetite (about 25%) must be noted and may be explained within the context of the high frequency of feeding problems [44]. However, it may be also associated with the underlying gastrointestinal disease as reduced appetite was described in other pediatric clinical samples as well [23].

With respect to demographic and anthropometric factors, the following were significantly associated with the presence of feeding problems: child’s age, only child, low zBMI, and low birth weight. More specifically, younger children were more likely to have feeding problems. A similar effect was demonstrated in children with eosinophilic gastrointestinal disorders in a previous study [22]. On the contrary, in reports of healthy children this finding was not reported [20,39,47]. One possible explanation for this finding would be that gastrointestinal diseases manifest early in the child’s life, sometimes even in infancy and gradually remit, usually completely, or are successfully treated as the child grows up. Since feeding problems are possibly due to gastrointestinal disease, it is anticipated that these problems will occur more often at younger ages when symptoms are more prominent or have not yet been adequately controlled with treatment. This finding suggests that families of younger children with gastrointestinal diseases may require the most support to manage feeding problems. Furthermore, being an only child was significantly related to abnormal TFS. This factor was found to influence parental feeding practices, sometimes leading to application of unfavorable strategies (e.g., distractions during meal) [18]. From another perspective, the absence of siblings and thus a lack of peer modeling may have negative consequences as peers hold an important role serving as models in inducing consumption of novel or previously disliked foods [48,49]. In addition, zBMI was negatively correlated with overall TFS and lower zBMI was associated with a higher probability of abnormal scoring. This might be possibly associated with the finding that TFS and TPS above the established cut-offs was concurrent with difficulties in gaining weight. These findings potentially demonstrate direct implications of feeding problems on nutrition and development as they have been previously described [2,50,51,52]. The nature of this study does not provide evidence for drawing causal conclusions. Otherwise stated, feeding problems may be due to low BMI or conversely, low BMI may be due to feeding problems. In any case, these are particularly important findings highlighting the serious impact of feeding problems on a child’s nutritional status and that, if left untreated, may lead to nutritional deficiencies [2,50,51,52]. The results of previous research aiming to investigate this relationship are contradictory, with several of them not finding any association between feeding behavior and nutrition [22,31,53,54]. Low birth weight has been already associated with feeding problems in premature children [55] and the same trend was demonstrated by the present study as low birth weight was related to a greater chance of abnormal BPFAS scores. Considering that prematurity was an exclusion criterion, the aforementioned association concerns children born full-term and is independent of prematurity and its consequences. In light of this, further studies are needed to investigate this relationship and to ascertain potential causes. Hypothetically, parents’ stance toward a child born underweight may lead to attitudes and behaviors that contribute to a negative nutritional environment. For instance, parents of low-birth-weight children with gastrointestinal diseases possibly make special effort to help their child maintain a normal weight that may probably lead to coercive practices which in turn lead to problematic feeding behavior.

Certain parameters of mealtime environment were also associated with BPFAS scores. It is noteworthy that the use of distractions during meals (bringing toys, use of screens) was correlated with abnormal BPFAS scores. The existing literature associates distraction during meals with feeding problems [43,56,57] or obesity in the pediatric population [58,59]. Parents often resort to this practice when their child does not eat and in order to gain weight they ignore the unfavorable impact it can have on child’s feeding behavior. Distractions have also been associated with higher energy intake and dietary habits of lower quality in preschool children [60]. It is also worth mentioning that the vast majority of the parental practices assessed in the present study were positively correlated with TFS and TPS indicating that these practices were very often applied by the parents of children with feeding problems, as the existing literature indicates [61,62,63,64]. This allows bidirectional interpretation; the association could either reflect the parents’ response to the preexisting child’s feeding problem or, on the contrary, may depict the contribution of parental practices on the onset and maintenance of a feeding problem. Practices that had the strongest correlation in the present study have been associated with adverse effects on food intake [13,64,65,66,67] and trying to limit them may prove beneficial. The present study thus highlights the urgent need of properly informing parents about the implementation of favorable practices or potential need for professional consultation with the aim of optimal management of the underlying problem. Results suggest that sitting at the table, about the same time every day and eating the same food together with the rest of the family shaped a favorable environment that was associated with appropriate feeding behaviors in the present study. Existing literature verifies that children who lack a structured and consistent meal routine are more likely to exhibit feeding problems [9,57,68,69]. In addition, as children learn to eat through observation and role-modeling by family members, this finding highlights the importance of systematically exposing the child to positive eating patterns and integrating into the feeding environment of the rest of the family [70]. This is particularly important for children with a gastrointestinal disease who in many cases have to adapt to specific dietary guidelines. Special diets, for example, may result in a child eating different food or having a separate meal program and thus may isolate the child from the family’s eating routine. Consequently, the child is deprived of the beneficial effects of role-modeling and structured family meal. In addition, the child’s behaviors that indicate ability to self-regulate food intake (expression of hunger), parental practices that encourage such behaviors (“I accept that he may not be hungry and I take the food away”) and autonomous eating were found to have favorable impact on the child’s feeding behavior in our study. Autonomous eating, in particular, has been negatively correlated to BPFAS scores emphasizing the significance of developing self-feeding. Previous studies have evaluated the positive effect of self-feeding as it is associated with lower selectivity rates during the introduction of solid foods [71]. Failure to develop self-feeding has been associated with the presence of feeding problems [43] and with non-IgE-mediated allergic gastrointestinal diseases [24]. On the contrary, ignoring signs of satiety (“I urge the child to eat through prompts”) had a positive correlation with abnormal BPFAS. These findings are consistent with existing literature highlighting the importance of the ability to self-regulate food intake and of parental practices that respect and respond to child’s hunger and satiety cues in order to contribute to the reduction of feeding problems [72,73,74]. Finally, correlation results provide the opportunity of forming potential indicators of feeding problems. More specifically, three questions (“the child eats autonomously”, “the child eats the same food as the rest of the family” and “I verbally urge the child to eat”) had the strongest correlation with both scores (TFS and TPS); therefore, they could be used toward this direction. From this perspective, these three questions could serve as a short and practical detecting questionnaire in pediatric population. The integration of such an extremely short but targeted screening tool into the routine of clinical practice is of significant importance, taking into consideration that it will not further burden the work of clinical pediatricians.

The high prevalence of feeding problems in children with gastrointestinal disease is an issue of major concern and requires further attention in order to ensure effective and efficient intervention. An important strength of the present study is that it provides multifaceted understanding of factors associated with feeding problems in children with gastrointestinal diseases. Another important strength is the use of the BPFAS, which is considered the most valid and reliable clinical measurement tool to detect feeding problems. Diagnosis of a feeding problem however requires clinical evaluation therefore results should be treated with caution. A limitation of our study is that inferences about causality cannot be safely drawn considering that cross-sectional studies do not provide such opportunity. Therefore, direction of the associations between feeding problems and other factors (anthropometric, demographic, environmental) is not possible. Interventional studies would be also essential in the scope of investigating causality between parenting practices and feeding problems. Assessment of mealtime environment and parental practices was conducted through a set of questions selected by the researchers and not through standardized questionnaires in order to assess multiple environmental factors related to feeding problems, avoid unnecessary repetition, and overlap with other questionnaires (BPFAS). This has contributed to identifying the most targeted questions that may form a future easy-to-use detection tool of feeding problems. In addition, although the voluntary participation of the parents in the study may introduce selection error, the response rate is high, providing reliable estimates. Similarly, the children were sampled from the Pediatric Gastroenterology Outpatient Clinic of one hospital, involving potential representation implications. Finally, future research should focus on subgroup analysis as each clinical group may form individual feeding characteristics and investigating those patterns may facilitate targeted guidance. When planning future studies, it is essential to investigate individual parameters of the treatment of each special disease.

## 5. Conclusions

In conclusion, the present study showed that parent-reported feeding problems are increased in children with gastrointestinal diseases. Certain parameters of mealtime environment and specific parental feeding practices were linked to feeding problems. These findings might direct future longitudinal studies to explore the direction of these associations. A multifaceted understanding of factors associated with feeding problems in this clinical population is motivational as it may be the first step toward a family based prevention program with varied perspectives and different types of expertise.

## Figures and Tables

**Table 1:** Diagnoses of the children with gastrointestinal diseases.

Diagnosis	Frequency (Percentage)
Food allergy enteropathy	67 (47.5%)
Food protein-induced Gastroesophageal Reflux Disease (GERD)	30 (21.3%)
Food protein-induced Enterocolitis Syndrome (FPIES)	27 (19.1%)
Gastroesophageal Reflux Disease (GERD)	14 (9.9%)
Celiac disease	3 (2.1%)

**Table 2:** Demographic and anthropometric characteristics of the sample and BPFAS scores.

		*N* (%)
Child sex	Male	77 (54.6)
Child age group	>5 yrs	99 (70.2)
Only child	yes	46 (32.6)
Firstborn	yes	91 (64.5)
Parental sex	Female	136 (96.5)
Parental age group	<40 yrs	109 (77.3)
Parental education	>12 yrs	92 (65.2)
Working parent	yes	84 (59.6)
TFS score by cut-off	>84	24 (18.6)
TPS score by cut-off	>9	47 (39.5)
Child’s age (years)	Mean ± sd Median (Q1, Q3)	4.2 ± 1.3 4.42 (3.33, 5.17)
BMI z-score (current)	Mean ± sd Median (Q1, Q3)	−0.2 ± 1.3 −0.29 (−1.08, 0.82)
Birth weight (grams)	Mean ± sd Median (Q1, Q3)	3212.1 ± 414.5 3160 (2960, 3500)
TFS score	Mean ± sd Median (Q1, Q3)	70 ± 17.9 67 (56, 79)
TPS score	Mean ± sd Median (Q1, Q3)	8.9 ± 6.75 8 (3, 14)

**Table 3:** Percentages of the answers on each item of BPFAS scale.

	TFS					TPS
	1 (Never)	2	3	4	5 (Always)	Perceived as Problem
1. Eats fruits	5.7	19.1	19.1	10.6	45.4	25.9
2. Has problems chewing food	70	17.1	4.3	5.7	2.9	14.4
3. Enjoys eating	0.7	10.7	20.7	40	27.9	24.8
4. Chokes or gags at mealtimes	78.4	15.8	4.3	1.4	0	13.9
5. Will try new foods	10	28.6	22.1	18.6	20.7	42.3
6. Eats meat and/or fish	5.9	7.4	13.2	13.2	60.3	22.2
7. Takes longer than 20 min to finish a meal	22.6	17.5	21.9	19	19	29.1
8. Drinks milk	19.3	7.9	13.6	10.7	48.6	20.3
9. Comes readily to mealtime	5	7.1	18.6	33.6	35.7	26.5
10. Eats junky snack foods but will not eat at mealtime	40	29.3	17.1	8.6	5	32.4
11. Vomits just before, at, or just after mealtime	95	4.3	0	0.7	0	2.9
12. Eats only ground, strained or soft food	73.6	7.9	4.3	6.4	7.9	12.4
13. Gets up from table during meal	27.1	30	20.7	14.3	7.9	41
14. Lets food sit in mouth and does not swallow it	60	24.3	9.3	5	1.4	26.6
15. Whines or cries at feeding time	52.1	27.1	13.6	4.3	2.9	30.7
16. Eats vegetables	9.3	14.3	20	27.9	28.6	35.1
17. Tantrums at mealtimes	45.3	36	14.4	2.9	1.4	26.7
18. Eats starches (for example potato noodles)	2.9	2.9	17.1	24.3	52.9	16.2
19. Has a poor appetite	32.1	30	13.6	14.3	10	38
20. Spits out food	57.9	30	7.9	2.1	2.1	18.8
21. Delays eating by talking	23.9	34.8	21.7	12.3	7.2	28.8
22. Would rather drink than eat	42.6	25.7	12.5	11	8.1	24.2
23. Refuses to eat meal, but requests food immediately after	57.9	22.9	12.1	4.3	2.9	24.1
24. Tries to negotiate what will or won’t eat	15	35	23.6	19.3	7.1	40.7
25. Has required supplemental tube feeds to maintain proper nutritional status	98.6	0	0.7	0.7	0	0
26. I get frustrated and/or anxious when feeding my child	49.6	18	12.9	12.9	6.5	41.6
27. I coax my child to get him/her to take a bite	35.7	26.4	12.1	17.9	7.9	34.8
28. I use threats to get my child to eat	55.7	25.7	11.4	7.1	0	27.9
29. I feel confident my child gets enough to eat	12.9	17.9	13.6	27.9	27.9	33.3
30. I feel confident in my ability to manage my child’s behavior at mealtime	7.1	12.1	19.3	22.9	38.6	28.7
31. If my child does not like what is being served, I make something else	33.6	34.3	10	13.6	8.6	35.6
32. When my child has refused to eat, I have put the food in his/her mouth by force If necessary	77.1	15.7	2.1	3.6	1.4	18.8
33. I disagree with other adults about how to feed my child	43.6	23.6	19.3	9.3	4.3	31.4
34. I feel that my child’s pattern hurts his/her general health	60.7	18.6	11.4	5	4.3	25.9
35. I get so angry with my child at mealtimes that it takes me a while to calm down after the meal	68.6	20	5.7	1.4	4.3	22.5

**Table 4:** Pathological BPFAS scores (TFS and TPS above the cut-offs) in association with the demographic and the anthropometric characteristics, the mealtime environment, and the parental practices.

	TFS	TPS
Demographic and anthropometric characteristics		
Child’s age	[−] **	[−] **
Child’s age > 5	[−] *	NA
Child’s zBMI	[−] *	[−] *
Birth weight	[−] *	[−] *
Only child	[+] *	NA
Poor weight gain	[+] ***	[+] **
Mealtime Environment		
We eat at the table	[−] ***	[−] *
We eat at least one meal a day all of us together (or almost all of us together)	[−] **	NA
My child eats the same food with the rest of the family	[−] ***	[−] *
My child eats at almost the same time every day	[−] **	NA
My child eats autonomously (without an adult’s help)	[−] ***	[−] ***
My child sits at the table during the meal	[−] ***	[−] **
My child brings toys (books, tablets, etc.) at the table during the meal	[+] **	[+] ***
My child informs me or conveys to me that he/she is hungry	[−] ***	[−] **
I allow my child to sit up from the table during the meal if he/she wishes to	[+] **	NA
Parental feeding Practices		
I accept that he/she may not be hungry, and I take the food away.	NA	[−] *
I urge the child to eat with prompts such as: “eat at least a little”, “please try to eat”, etc.	[+] ***	[+] ***
I ask my family or other people to encourage the child to eat.	[+] ***	[+] ***
I say to my child that I or someone else in the family is eating	[+] ***	[+] ***
I feed my child myself to make him/her eat his/her food.	[+] ***	[+] *
I help my child eat the food.	[+] ***	[+] *
I move to a different feeding area	[+] ***	[+] *
I customize the environment so that the child can eat.	[+] ***	[+] ***
I say to the child “if you don’t eat, I’ll be sad”.	NA	[+] *
I offer in exchange for the food a game or activity	[+] ***	[+] **
I offer some other food in exchange for the meal.	NA	[+] *
I say something to show my displeasure when the child is not eating.	[+] ***	[+] *
I have to make a physical effort to make the child eat.	[+] ***	[+] *

Note: *: 0.01 < *p* < 0.05; **: 0.001 < *p* < 0.01; ***: *p* < 0.001; *p* values for Mann–Whitney U test or chi-square tests depending on the type of the variables. [+]: positive association, [−]: negative association, NA: no association.

## Data Availability

The data presented in this study are available within the article.
